# Chronic Giardiasis: Devil of a developing country

**DOI:** 10.12669/pjms.41.12.12123

**Published:** 2025-12

**Authors:** Punhal Khan, Mian Shah Yousaf, Lubna Kamani, Rabia Ali

**Affiliations:** 1 Punhal Khan, *MBBS, FCPS*. Liaquat National Hospital, Karachi, Pakistan; 2 Mian Shah Yousaf, *MBBS, FCPS*. Prime Teaching Hospital, Peshawar, Pakistan; 3 Lubna Kamani, *MBBS, FCPS*. Liaquat National Hospital, Karachi, Pakistan; 4 Rabia Ali, *MBBS*. Liaquat National Hospital, Karachi, Pakistan

**Keywords:** Gastroptosis, Giardiasis

## Abstract

Gastroptosis is a clinical condition in which the stomach is displaced downward from its normal position in the belly. The reasons for its present prevalence are not clear due to the availability of scarce medical literature. Giardia affects almost two hundred million people per annum, making it the most common intestinal parasite. In this paper, we report on a 30 years old female who presented with abdominal pain, vomiting, weight loss, and loose stools and was diagnosed with gastroptosis on a barium meal. The diagnosis of giardiasis was established based on a duodenal biopsy, and the patient was followed till complete resolution of symptoms after treatment.

## INTRODUCTION

Gastroptosis is an anatomical condition with a downward displacement of the stomach, in which the greater curve of the stomach is dislocated below the iliac crest. It is diagnosed by using imaging studies of the belly in a standing position.^1^ Gastroptosis can cause abdominal discomfort, epigastric pain, loss of appetite, early satiety, or even gastric emptying disorders. These symptoms are usually aggravated with food intake, and the common differential diagnosis includes peptic ulcer disease, pancreatitis, and gastritis.^1^,^2^ However, a few anatomic conditions, such as annular pancreas, congenital stenosis of the duodenum, intestinal malrotation, or pancreatic tumors, should also be included in differentials.^3^ Gastroptosis can impact the quality of life badly because the symptoms get postprandial aggravation, which can then disrupt daily activities.

Giardia lamblia (also referred to as Giardia intestinalis and Giardia duodenalis) is known to be the most common intestinal parasite, most commonly found in developing countries. It is a flagellated microorganism belonging to the genus Giardia and colonizes the small bowel, leading to chronic diarrhea, also called giardiasis. Giardia is transmitted by the fecal-oral route, mostly by ingestion of contaminated water or food. Signs and symptoms mostly appear two to four weeks post-exposure and include large-volume watery and foul-smelling diarrhea, abdominal pain, flatulence, bloating, and nausea. Giardia lamblia clears without any treatment in most immunocompetent people; however, in certain cases, it can lead to failure to thrive, malnutrition, and growth retardation. Undernourished children and immune-deficient individuals from the developing world are more susceptible to serious complications of untreated giardiasis. Due to contaminated water supply and poor sanitation, the prevalence of giardiasis in developing countries is estimated to be around 20% to 30%.^4^ Prevalence is estimated to be 2% to 5% in industrialized countries. Giardia should be considered in differentials of a lot of GI diseases especially in high prevalence areas.

## CASE PRESENTATION

**Fig.1:**
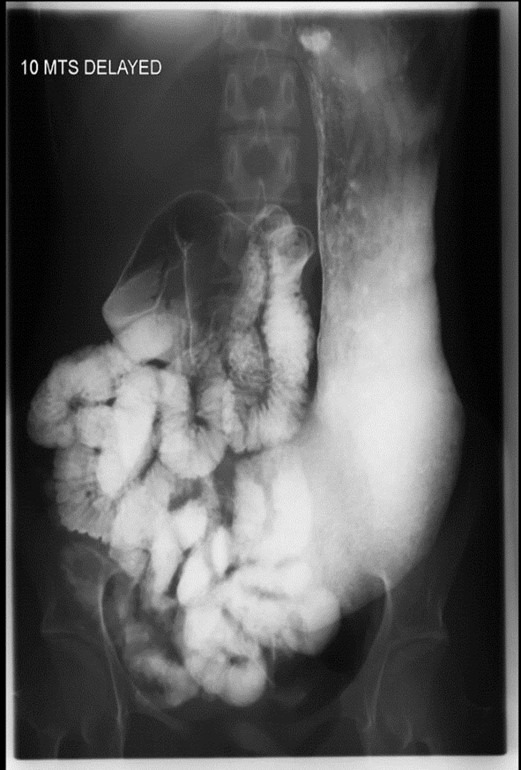
Barium.

**Fig.2:**
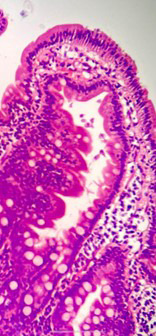
Histology.

A 30 years old married female resident of Karachi, Pakistan, presented in the outpatient gastroenterology department with complaints of abdominal pain, loose stools, intermittent bouts of vomiting, and bulky loose stools for two years. She lost 15 kilograms of weight over two years. She denied any history of illicit drugs or herbal medicines. Detailed history and examination were done along with laboratory and radiological tests. Her blood workup showed hemoglobin of 10 g/l with low serum iron, ferritin, vitamin B12, and vitamin D. Liver function tests, urea, creatinine, electrolytes, and a detailed stool report were all normal. A barium follow-through study (Fig.1) shows a distended and elongated stomach appearance that represents gastroptosis and also suggests hypermobility of the bowel. CT scans done for weight loss also revealed similar findings. Gastroscopy and biopsy were performed, and duodenal biopsies showed Giardia lamblia (Fig.2). The patient was given a single dose of two grams of tinidazole with supportive and symptomatic therapy. A nutrition consult was also taken for a caloric diet. After six months of treatment and a high-caloric diet, the patient regained eight kg of weight, and all symptoms subsided.

## DISCUSSION

Visceroptosis is also known as Glenard’s disease; it is a condition in which abdominal organs are displaced below their natural position. When visceroptosis involves the stomach, then the condition is referred to as gastroptosis. Gastroptosis is diagnosed on imaging when the stomach is seen to be displaced downwards, having its greater curve projected below the iliac crest, while the antrum remains in its normal position.^5^ Gastroptosis was first reported in 1833 by Glenard. After that, only a few cases have been reported. All the available data shows that women between the ages of 20 and 50 years are mostly affected by gastroptosis, with high risk in those who have postural defects and low weight.^6^

It is mostly caused by the abdominal wall laxity, which leads to thinning and relaxation of the mesenteric attachments of the stomach under the organ’s weight. In addition to this, another predisposing factor is a decreased amount of visceral fat within the lesser omentum.^7^ Gastroptosis has no specific symptoms, but the most common presentations of this condition include epigastric discomfort or pain, nausea, early satiety, vomiting, and flatulence, and all these symptoms are aggravated during post-meal activity and in the standing position.^7^,^8^ The main diagnostic tool remains to be the upper GI tract barium or gastrograffin studies. Other common causes of gastroparesis, such as diabetes, hypothyroidism, viral infections, and muscular dystrophy, should be ruled out before fluoroscopy.

Differential diagnoses of gastroptosis should include celiac disease, inflammatory bowel diseases, GERD, and various disorders of pancreaticobiliary origin. Furthermore, gastroparesis and bowel obstruction may accompany gastroptosis, so that should always be taken into consideration. In Glenard’s times, the usual approach for such patients was to do surgical restoration.^6^ But nowadays, only those cases that are complicated by bowel obstruction are treated invasively.^9^ Other treatment options include prokinetic medicines and adequate dietary measures. Physiotherapy also plays an important role, especially focused on paraspinal and abdominal muscle strengthening.

As per the Infectious Disease Society of America (IDSA) diagnostic guidelines, Giardia duodenalis is commonly diagnosed by nucleic acid amplification testing (NAAT) or stool antigen test. When clinical suspicion of giardia is high and stool tests are non-diagnostic, then duodenal aspirate microscopy is recommended by IDSA guidelines. A duodenal biopsy is not a common diagnostic tool for giardiasis. In our case, it was diagnosed on duodenal biopsy, and the patient was treated accordingly. In our opinion, chronic giardiasis in our patient leads to significant weight loss, which then predisposes our patient to gastroptosis.

In recent times gastroptosis has rarely been reported. In our opinion, this is the first reported case of giardiasis leading to gastroptosis. An important learning lesson, in this case, is that gastroptosis and other motility disorders can co-exist with chronic giardiasis and should be taken into consideration when the patient is not responding to routine treatment.

### Author’s Contributions:

**PK:** Conception and design of work, data acquisition, data interpretation, manuscript preparation.

**MSY:** Data acquisition, data interpretation, provided subject matter expertise and is responsible for its final content and is the article guarantor.

**LK and RA:** Conception and design of work, data interpretation, manuscript revision.

All authors have read and approved the final version of the manuscript.

## References

[1] Bestari MB, Chandra M, Joewono IR, Girawan D, Andhika R, Wahyudi Y (2022). Gastroptosis due to gastric outlet obstruction secondary to duodenal tumour: Glenard's disease revisited. Case Rep Gastroenterol.

[2] Staszewska A, Jarzumbek A, Saran A, Gierak-Firszt S, Kwiecien J (2023). Postprandial Abdominal Pain Caused by Gastroptosis-A Case Report. Children (Basel).

[3] Fernandes ND, Murphy SA (2021). Gastroptosis: An Uncommon Cause for a Deep Nasogastric Tube. Clin Gastroenterol Hepatol.

[4] Hassan SM, Mubarik A, Muddassir S, Haq F (2018). Adult idiopathic hypertrophic pyloric stenosis - a common presentation with an uncommon diagnosis. J Community Hosp Intern Med Perspect.

[5] Van Welie AJM, Klein WM, Draaisma JMT (2017). The clinical or radiographic diagnosis of gastroptosis: Still relevant?. Gastro Open J.

[6] Ali R, Yaniv R, Richard S, Xiaochen L (2018). Ehlers-Danlos Syndrome Type III (EDS) and Visceroptosis: Getting to the Bottom of This Diagnosis. Am J Gastroenterol.

[7] Sigurdsson L, Flores A, Putnam PE, Hyman PE, Di Lorenzo C (1997). Postviral gastroparesis: presentation, treatment and outcome. J Pediatr.

[8] Christianakis E, Bouchra K, Koliatou A, Paschalidis N, Filippou D (2009). Gastroparesis associated with gastroptosis presenting as a lower abdominal bulking mass in a child: a case report. Cases J.

[9] Natsis K, Apostolidis S, Papadopoulou AL, Vlasis K, Totlis T, Skandalakis P (2008). Gastric femoral hernia in a male cadaver with gastroptosis: case report and review of the literature. Hernia.

